# The SARS-CoV-2 receptor ACE2 expression of maternal-fetal interface and fetal organs by single-cell transcriptome study

**DOI:** 10.1371/journal.pone.0230295

**Published:** 2020-04-16

**Authors:** Mengmeng Li, Liang Chen, Jingxiao Zhang, Chenglong Xiong, Xiangjie Li

**Affiliations:** 1 Department of Obstetrics and Gynecology, Peking Union Medical College Hospital, Peking Union Medical College, Chinese Academy of Medical Sciences, Beijing, China; 2 Department of Epidemiology, School of Public Health, Fudan University, Shanghai, China; 3 Center for Applied Statistics, School of Statistics, Renmin University of China, Beijing, China; 4 School of Public Health, Key Laboratory of Public Health Safety, Ministry of Education, Fudan University, Shanghai, China; Chinese University of Hong Kong, HONG KONG

## Abstract

The new type of pneumonia caused by the SARS-CoV-2 (Severe acute respiratory syndrome coronavirus 2) has been declared as a global public health concern by WHO. As of April 3, 2020, more than 1,000,000 human infections have been diagnosed around the world, which exhibited apparent person-to-person transmission characteristics of this virus. The capacity of vertical transmission in SARS-CoV-2 remains controversial recently. Angiotensin-converting enzyme 2 (ACE2) is now confirmed as the receptor of SARS-CoV-2 and plays essential roles in human infection and transmission. In present study, we collected the online available single-cell RNA sequencing (scRNA-seq) data to evaluate the cell specific expression of ACE2 in maternal-fetal interface as well as in multiple fetal organs. Our results revealed that ACE2 was highly expressed in maternal-fetal interface cells including stromal cells and perivascular cells of decidua, and cytotrophoblast and syncytiotrophoblast in placenta. Meanwhile, ACE2 was also expressed in specific cell types of human fetal heart, liver and lung, but not in kidney. And in a study containing series fetal and post-natal mouse lung, we observed ACE2 was dynamically changed over the time, and ACE2 was extremely high in neonatal mice at post-natal day 1~3. In summary, this study revealed that the SARS-CoV-2 receptor was widely spread in specific cell types of maternal-fetal interface and fetal organs. And thus, both the vertical transmission and the placenta dysfunction/abortion caused by SARS-CoV-2 need to be further carefully investigated in clinical practice.

## Introduction

The new type of pneumonia caused by the SARS-CoV-2 has sparked alarm around the world.[1] The ongoing outbreak was first reported in Wuhan, China, in December 2019 and as of April 3, 2020 more than 1,000,000 human infections have been confirmed around the world.[2] Person-to-person transmission has been described both in hospital and family settings. [3] To date, there are no effective drugs or vaccination available against SARS-CoV-2.

Notably, SARS-CoV-2 shared 79% sequence identify to SARS-CoV (Severe acute respiratory syndrome coronavirus) and they may both share the ACE2 as host receptor according to structural analyses.[4] SARS-CoV uses ACE2 as one of the main receptors for the entry into the host cells which plays a crucial role in the disease infection.[5] The target towards the interaction between the virus and receptor may be able to treat the disease. ACE2 is newly described as Renin-angiotensin system (RAS) component and modulates blood pressure.[6] The expression and distribution of ACE2 has been reported in heart, lungs and kidneys, which exhibits tissue-specific activity patterns.[7–9] Previous studies have also shown the expression of ACE2 in the placenta.[10] Furthermore, the serine protease for virus Spike (S) protein priming, TMPRSS2, was identified to be indispensable for cell entry of SARS-CoV-2. [11]

The placenta is a unique mixed organ, acting as heart, lungs, liver, kidneys for the fetus, which is formed only during pregnancy and plays a major role in preventing maternal-fetal transmission of pathogens.[12] It has been reported that members of the coronavirus family such as SARS-CoV and Middle East respiratory syndrome (MERS-CoV) may pose greater risk in pregnant women than non-pregnant individuals and are responsible for severe complications during pregnancy.[13, 14] Considering the new SARS-CoV-2 seems to share similar pathogenic and cell receptor as SARS-CoV, the new coronavirus may have the vertical transmission potential to the fetus in pregnant women with SARS-CoV-2. [15]

Given the current lack of clinical data of the potential and outcome of pregnancy infected by the SARS-CoV2, we use the promising scRNA-seq data to evaluate the expression of ACE2 and TMPRSS2 in maternal-fetal interface and different fetal organs. Our study gives a better perspective of vertical transmission potential and virus’s impact on placenta function and early pregnancy on the cellular level.

## Method

### Public dataset acquisition and processing

**Fig 1:**
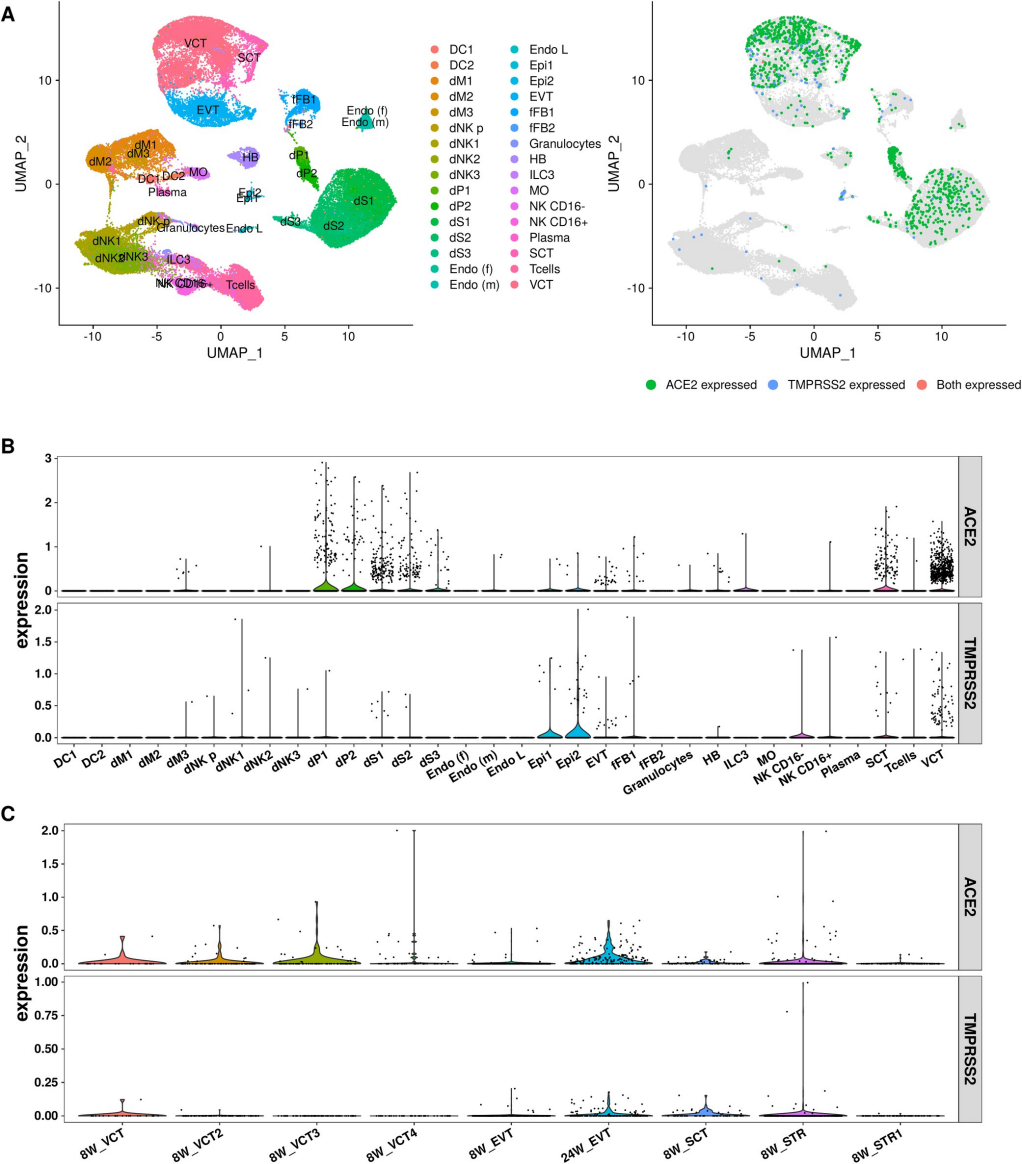
The expression level of ACE2 and TMPRSS2 in human placenta. (A)-(B) are the results from Vento-Tormo, R et al.(2018).[16] (A) The tSNE plots of cell types and ACE2/TMPRSS2 genes. For the right plot, the points colored red are the cells expressed ACE2.(B)The violin plot of ACE2 gene in difference cell types. (C) The expression level of ACE2/TMPRSS2 for the data set from Liu, et al.(2018). DC: dendritic cells; **dM**: decidual macrophages; **dP**: decidual perivascular cells; **dS**: decidual stromal cells; **Endo**: endothelial cells; **Epi**: epithelial glandular cells; **FB**: fibroblasts; **HB**: Hofbauer cells; **PV**: perivascular cells; **SCT**: syncytiotrophoblast; **VCT**:villous cytotrophoblast; **EVT**: extravillous trophoblast; **CTBs**: cytotrophoblast cells; **EVTs**: extravillous trophoblast cells; **STR**: villous stromal cells.

Human placenta: Gene expression matrix and the cell type annotation of scRNA-seq of the early maternal-fetal interface in human can be downloaded from E-MTAB-6701 (corresponding to Fig 1A and 1B).[16] And another dataset of human placenta can be download by the Gene Expression Omnibus GSE89497 (corresponding to Fig 1C).[17]

Human fetal Heart: Gene expression matrix of scRNA-seq data of human fetal heart was downloaded from the Gene Expression Omnibus (GSE106118) and the annotation was downloaded from the supplementary table1 from the Cui, et al (2019).[18]

Human fetal liver: Gene expression matrix of scRNA-seq data of human fetal liver was downloaded from ArrayExpress with accession code E-MTAB-7407 and the annotation was downloaded from the supplementary table from Popescu, et al.(2019).[19]

Human fetal kidney: The dataset can be extracted based on the dataset downloaded from E-MTAB-7407.

Human Lung: The average expression of ACE2 and TMPRSS2 across different cell types for Human lung with post-natal day (PND1) can be downloaded from Lung Gene Expression Analysis Web Portal (https://research.cchmc.org/pbge/lunggens/genequery_dp.html?spe=HU&tps=pnd1&geneid=ace2).

Mouse fetal Lung: The expression level of ACE2 and TMPRSS2, the p.value and corresponding fold change for Mouse lung at E16.5, E18.5, PND1, PND3, PND7, PND10, PND15 and PND28 can be downloaded from Lung Gene Expression Analysis Web Portal (https://research.cchmc.org/pbge/lunggens/genequery_dp.html?spe=HU&tps=pnd1&geneid=ace2).

### Processing and visualization of scRNA-seq data

The downloaded unique molecular identifier (UMI) count matrix was converted to Seurat object using the R package Seurat v.3.1.1.[20] Then we normalized the raw gene expression matrix using NormalizeData function with default parameters and visualized the expression level using the Violin plot function in Seurat. For the Human placenta, we used the standard pipeline of Seurat https://satijalab.org/seurat/v3.1/pbmc3k_tutorial.html. Since the low quality cells had been excluded by original authors, we didn’t conduct the quality control procedures.

## Results

### Cell specific expression of ACE2/TMPRSS2 in maternal-fetal interface

Placenta and decidua are the main maternal-fetal interface during pregnancy, and virus receptors expression in placenta and decidual cells may play important role in promoting transmission of SARS-CoV-2. We obtained single cell transcriptome data sequenced by 10X Genomics of early placenta (6~14 gestational weeks) containing ~65,000 cells.[16] And 32 cell types were observed, of which four main cell types expressed ACE2 gene, including stromal cells (dS) and perivascular cells (dP) in decidua, and villous cytotrophoblast (VCT) and syncytiotrophoblast (SCT) in placenta (**Fig 1A and 1B**). The extravillous trophoblast (EVT) did not express ACE2 at this time. TMPRSS2 was expressed in VCT and epithelial glandular cells (Epi), and also had low expression in SCT. Another independent single cell study of trophoblasts in human placenta confirmed the expression of ACE2 and TMPRSS2 in VCT and SCT (**Fig 1C**).[17] In addition, the EVT cells had extremely low level of ACE2 at early placenta (8 week) which was consistent with previous study, while the ACE2 expression was significantly increased in EVT at later stage of pregnancy (24 week). And TMPRSS2 also found a similar dynamic alteration (**Fig 1C**). These results suggest that ACE2 and TMPRSS2 were co-expressed in VCT, SCT and EVT cells in maternal-fetal interface, and the expression level of ACE2/TMPRSS2 in maternal-fetal interface may increase along with trimester of pregnancy.

### Cell specific expression of ACE2/TMPRSS2 in human fetal organs

**Fig 2:**
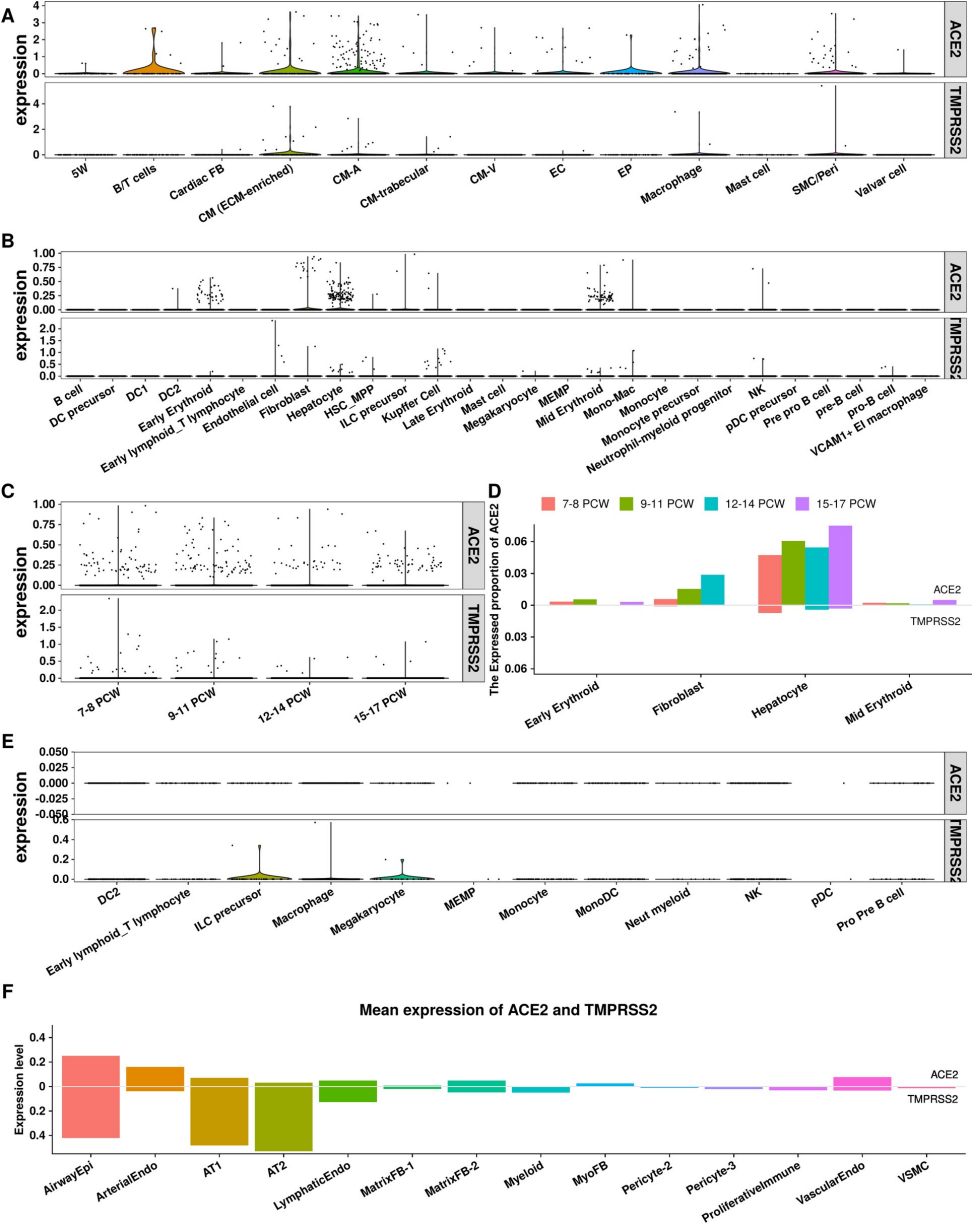
The expression level in different organs. (A) The expression of ACE2/TMPRSS2 in Human fetal Heart. **CM-A:** Atrial CM cells; **CM-V**: Ventricle CM cells, **EC**: Endothelial cdlls; **EP**: Epithelial cells; **SMC**: smooth muscle cells; **Peri**: Pericyte cells; **5W**: 5 weeks cells. (B) The expression of ACE2 across different cell types in Human fetal liver. **MEMP**: mega-karyocyte–erythroid–mast cell progenitor; **DC**: dendritic cells. **NK**: Nature killer cells (C) The expression of ACE2/TMPRSS2 across different PCW (post-conception weeks) in Human fetal liver. (D) The expression proportion of ACE2/TMPRSS2 across different PCWs for the most expressed four cell types (early Erythroid, Fibroblast, Hepatocyte, Mid Erythroid) in Human fetal liver. (E) The expression ACE2 in Human fetal kidney. (F) The average expression level of ACE2/TMPRSS2 in Human lung at post-natal day 1 (PND1). **AT1**/**AT2**: alveolar type 1/2 cells.

As the essential elements of virus transmission, the expression of virus receptors in target organs is another determining factor for fetus vulnerable to SARS-CoV-2. We then screened the ACE2 and TMPRSS2 expression in multiple fetal organs including heart, lungs, liver and kidneys based on the online published single cell transcriptome studies. In a fetal heart single cell study covering early to late fetal stages [18], we observed that ACE2 was expressed in cardiomyocytes (CM), macrophages and smooth muscle cells and pericytes (SMC/Peri). TMPRSS2 was also observed in cardiomyocytes. In a human fetal liver cell atlas [19], ACE2 was detected in erythroid, fibroblast and hepatocyte (**Fig 2B**). Although it seems that there was no change of ACE2 over the gestational stage (**Fig 2C**), the expression proportion of ACE2 was gradually increased in fibroblast and hepatocytes from early to mid-stage of pregnancy. (**Fig 2D**) And hepatocytes possessed the highest proportion of ACE2 positive cells compared to fibroblast and erythroid. (**Fig 2D**) The expression of TMPRSS2 was relatively lower, but could be found in hepatocyte and kuppffer cells. (**Fig 2B**) In a single cell study of fetal kidney at 7–9 week, we did not observe ACE2 expression in any cell type; and the abundance of TMPRSS2 was extremely low, and there were only few positive cells in ILC precursor and megakaryocyte subtypes. (**Fig 2E**) In a lung cell atlas of human at post-natal day 1, we observed that both ACE2 and TMPRSS2 were highly expressed in airway epithelial cell and arterial endothelial cells (AT1 and AT2) (**Fig 2F**). As lung is the major target organ of SARS-CoV-2, the high co-expression of ACE2 and TMPRSS2 strongly suggests a high risk of infection of newborns through airborne transmission. These data showed the evidence of abundant expression of ACE2 and moderate expression of TMPRSS2 in the main human fetal organs.

### Dynamic expression of ACE2/TMPRSS2 in fetal and neonatal mouse lung

**Fig 3:**
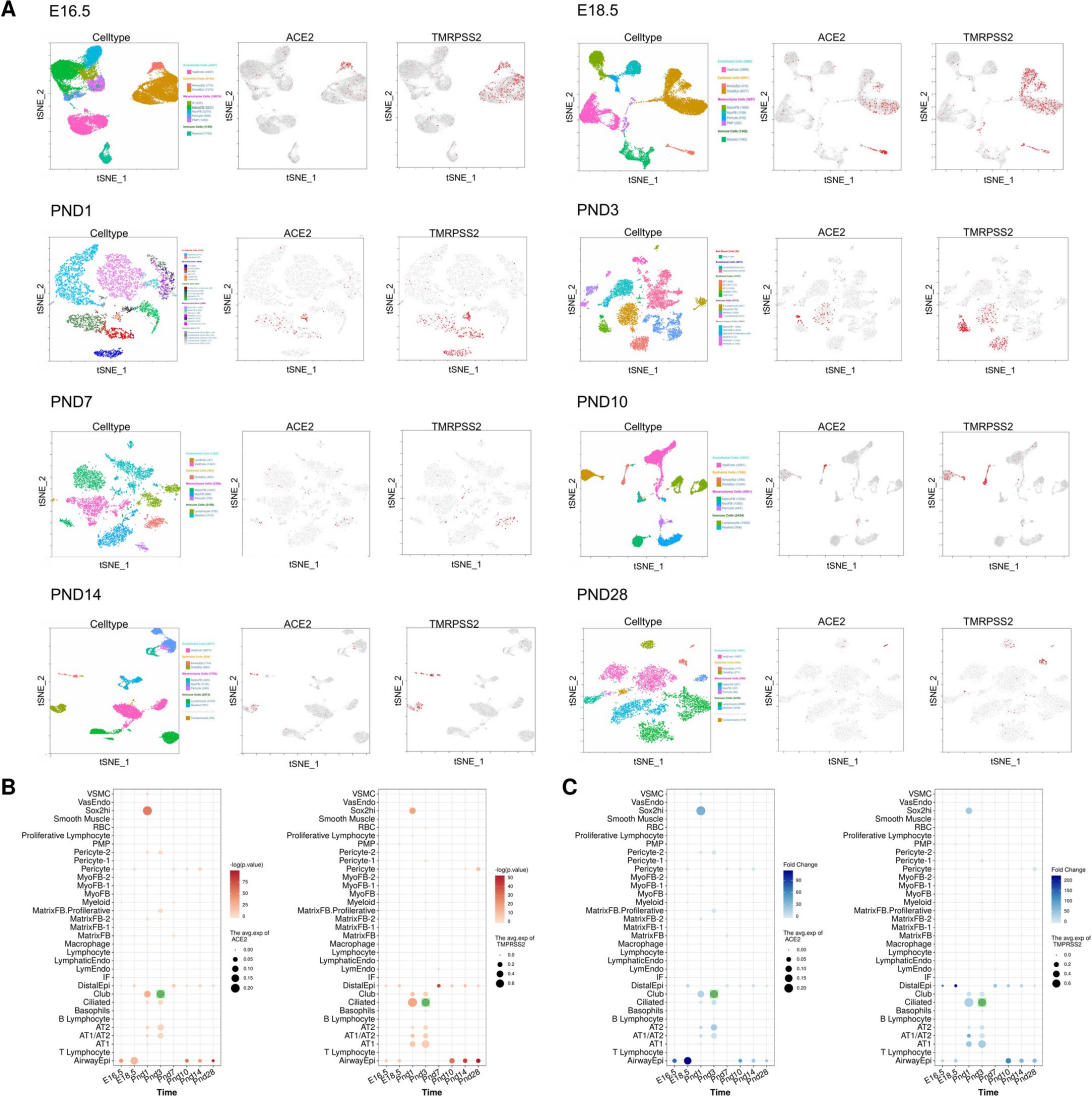
The expression level of ACE2/TMPRSS2 in mouse fetal lung (E16.5, E18.5, PND1, PND3, PND7, PND10, PND15 and PND28). (A) The dot plot of ACE2/TMPRSS2 in different time across different cell types. The point size represents the average level of ACE2/TMPRSS2 and the color scale means the -log(p.value). The expression level ACE2/TMPRSS2 for the point marked by green color is about 0.867. (B)The dot plot of ACE2 in different time across different cell types. The point size represents the average level of ACE2 and the color scale means the fold change. (C)The tSNE plots of cell type and feature plots of ACE2/TMPRSS2 in different times.

Since the respiratory system undergoes a series of structural and functional changes necessary for adaptation to air breathing at birth, and lung is a major organ to be attacked by SARS-CoV-2 virus, the dynamic changes of pulmonary cells gene expression are necessary to be investigated before and after birth to predict the potential infection of fetal and neonatal individuals. Because cell atlas of human fetal lung is unavailable, we obtained mouse lung cell atlas from late pregnancy (E16.5, E18.5) to PND 28. The cell type annotation obtained from previous report was shown in **Fig 3A**.[21] In late pregnancy stage, both ACE2 and TMPRSS2 were highly expressed in airway epithelial cells, which were consistent with human fetal lung. A significant alteration of ACE2 and TMPRSS2 was observed in murine lung. At earliest days (PND 1~3), we observed ACE2/TMPRSS2 was highly co-expressed in many cell types such as Sox2hi, alveolar cells (AT1/AT2), ciliated cell and Club cells. Pulmonary cells at this stage exhibited higher expression of ACE2 than that in fetal phase or later days, suggesting that newborn might be a high-risk population vulnerable to be infected by SARS-CoV-2. After the PND 1~3, ACE2 and TMPRSS2 recovered to relatively low level in lung and mainly expressed in epithelial cells, which is similar to that in adult human lung [22] (**Fig 3B and 3C**). These results suggest that the alternative expression of ACE2/TMPRSS2 in pulmonary cells before and after birth may contribute to the virus infection through vertical or respiratory transmission, and ACE2 and TMPRSS2 were highly co-expressed across different cell types of murine lung. And the molecular cell study of human lung at fetal and post-natal stage is further necessary, to reveal the potency and mechanism of infant infection by SARS-CoV-2.

## Discussion

As the SARS-CoV-2 pandemic continues, pregnant women may be at high risks of affection due to under an immunosuppressive state, and the affection status of mothers may cause adverse maternal and neonatal complications and outcomes.[15] Following this, the urgent questions need to be addressed are that whether the SARS-CoV-2 could be transmitted vertically to the fetus through maternal-fetal interface and whether the affection of the virus would cause the dysfunction of placenta or even abortion among pregnant women.

Based on the scRNA-seq data of early human placenta (first trimester), we have identified the high expression of ACE2 in four main cell types including dS cells and dP cells in decidua, VCT and SCT cells in placenta. However, the expression of ACE2 was at extremely low level in EVT at first trimester. The second study of human placenta confirmed the result and showed increasing expression of ACE2 in EVT at 24 week. The major function of the placenta is maintained by trophoblast cells including VCT, SCT and EVT.[23] The SCT is the outer lining of the placental villi which has direct contact with maternal blood flowing into the intervillous space which also plays as the main site of maternal and fetal exchange.[24] The high expression of ACE2 in these cells suggests that placenta has the potential to be infected by SARS-CoV-2 and may cause placenta dysfunction and pregnancy complications. Yet there is no scRNA-seq data human placenta in third trimester, we predict the ACE2 and TMPRSS2 expression in maternal-fetal interface may increase along with trimester of pregnancy. Importantly, it has also been validated the abundant existence of protein expression of ACE2 in the dS cells in decidua and SCT cells in placenta using immunohistochemistry (IHC), which was in accordance with the mRNA expression pattern from our findings.[10, 25]

We then assessed the expression of ACE2 and TMPRSS2 in the main organs including fetal heart, lung, liver and kidney. The expression of ACE2 was detected in the main cell types of heart like CM and SMC/Peri, and TMPRSS2 was also observed in CM. CM is the major functional cells of heart, and may cause cardiac dysfunction if affected by virus.[26] SMC/Peri cells are major perivascular cell types [27], and may be involved in the implantation of virus through infiltration of blood vessels. The increasing expression of ACE2 in liver fibroblast and hepatocytes was detected from first to second trimester. These results suggest that fetal liver is an vulnerable target organ of SARS-COV-2 virus, and its risk is increasing as the pregnancy progresses. Interestingly, we did not detect the ACE2 expression in kidney while TMPRSS2 was moderately expressed in this organ. This result is different from adult human kidney in which ACE2 is highly expressed in proximal convoluted tubule cells and proximal tubule cells.[28] We further analyzed the data of human lung at PND 1, both airway epithelial cell and arterial endothelial cells showed high expression of ACE2. This result is different from adult lung in which alveolar cells (AT1/AT2) were the major ACE2+ cell types. [22] As the fetus has no breath in maternal uterus, the airway epithelial cell may not be responsible for the vertical transmission of virus, but may play essential role in neonatal infection via respiratory transmission. Alternatively, the arterial endothelial cell expression of ACE2 may contribute to the potential intrauterine infection of fetal lung. The data of a study containing series fetal and post-natal mouse lung was analyzed, and we observed ACE2 and TMPRSS2 were extremely high in neonatal mice at PND 1~3. The high level of ACE2 in lung may make neonate vulnerable target of SARS-CoV-2. Humans and mouse are highly conservative regarding gene expression pattern, and we speculate that ACE2 may have the similar dynamic alterations of gene expression in human lungs from fetal to neonatal phase, which needs to be further investigated to evaluate the risk of intrauterine infection, canal infection and transmission possibility through postnatal respiratory systems.

Given the genomic similarity between SARS-CoV-2 and SARS-CoV, many clinical studies have done on pregnant women affected by SARS-CoV. To our knowledge, no published articles have reported the existence of maternal-fetal transmission of SARS-CoV in clinical cases.[29] Considering the low spread ability of SARS-CoV with ~8,000 infected patients worldwide, the population of infected pregnant women was minimal among them, and thus the potency of vertical transmission was evaluated insufficiently in SARS. However, the SARS-CoV-2 has become super-spreading event around the world and been announced as a pandemic by WHO, which caused more than 1,000,000 cases worldwide as of April 3, 2020. Much more cases of pregnant women infected by SARS-CoV-2 have been observed, compared to SARS-CoV. Chen, et al has recently reported the result of pregnancy of nine pregnant women who had pneumonia caused by SARS-CoV2.[30] Four infants were born prematurely but none were born earlier than 36 weeks of gestation. All the infants and samples including amniotic fluid, cord blood, neonatal throat swabs showed negative result of the virus. However, given the small sample size, the short time between illness onset and delivery and the biased included pregnant women who were all at the late-stage of pregnancy and gave birth by cesarean section, vertical transmission of SARS-CoV-2 still cannot be ruled out in this study. Most recently, Dong et al. has reported a newborn with elevated IgM antibodies to SARS-CoV-2. As IgM cannot be transferred across the placenta, and can only be produced by the fetus in response to virus, this result suggests that the neonate was infected in utero. And the liver injury of this neonate from the laboratory results also indirectly supports the possibility of vertical transmission.[31] Another report collected the data of 6 pregnant women with SARS-CoV-2. Notably, the virus-specific antibodies were detected in neonatal blood sera samples and the IgG concentrations which could be passively transferred across the placenta from mother to fetus elevated in 5 infants. Importantly, 2 infants were also detected the existence of IgM.[32, 33] These clinical studies strongly support the potency of vertical transmission of SARS-CoV-2, which is highly consistent with our hypothesis based on the cell specific enriched expression of the virus receptors in maternal-internal face and fetal organs.

Furthermore, women infected by SARS-CoV during pregnancy had a higher incidence of adverse outcomes including spontaneous miscarriage, premature delivery, and intrauterine growth restriction.[13] High maternal morbidity and mortality was associated with SARS-CoV during pregnancy.[34]. The placental pathophysiology changes may be correlated with clinical courses of the mother and fetus.[35] But the number of reported cases of pregnant women with SARS-CoV is very limited. Nevertheless, pregnant women with SARS-CoV-2 had mild symptoms and better outcome compared with those with SARS-CoV.[30] The biophysical and structural evidence showed that SARS-CoV-2 bind ACE2 with higher affinity than SARS-CoV, which suggested that SARS-CoV-2 might have more possibility to attack placenta[36] The published cases so far are quite limited and are restricted to women developing SARS-CoV-2 infection in third trimester (late pregnancy stage). It is still crucial to pay special attention to pregnant women infected with SARS-CoV-2 in early stage, during which the virus may affect the function of placenta and increase the risk of miscarriage.

### Study limitations

There are several limitations to this study. First, the gene expression levels were detected at mRNA levels. Although several previous studies confirmed the protein expression of ACE2 in placenta by IHC, systematic experimental validation of the protein distributions in maternal-fetal interface and fetal organs is necessary in the future. The second, this study reported the distribution of ACE2 and TMPRSS2, but the existence of vertical transmission and placenta dysfunction caused by SARS-CoV-2 infection is just speculative, which needs to be further evaluated by clinical investigations. The third, we revealed the dramatic alterations of ACE2/TMPRSS2 expression from fetal to neonatal phases in mouse lung, which may contribute to the respiratory transmission in neonates. But the alterative gene expression pattern in human lung is not available to date, which needs to be further illuminated in the future.

## Conclusion

This study demonstrates the expression of SARS-CoV-2 receptors in human maternal-fetal interface and the main fetal organs. Both the vertical transmission and the placenta dysfunction/abortion caused by SARS-CoV-2 need to be further carefully investigated in clinical practice.
